# Increasing the use of continuing professional development courses to strengthen trauma care in Ghana

**DOI:** 10.4314/gmj.v54i3.11

**Published:** 2020-09

**Authors:** Samuel Debrah, Peter Donkor, Charles Mock, Joseph Bonney, George Oduro, Michael Ohene-Yeboah, Robert Quansah, Stephen Tabiri

**Affiliations:** 1 Department of Surgery, University of Cape Coast School of Medical Sciences, Cape Coast; 2 Department of Surgery, Kwame Nkrumah University of Science and Technology, P. O. Box 1934, Kumasi; 3 Department of Surgery, University of Washington, Box 359960, 325 Ninth Avenue, Seattle, WA, USA; 4 Directorate of Emergency Medicine, Komfo Anokye Teaching Hospital, P. O. Box 1934, Kumasi; 5 College of Health Sciences, Department of Surgery, University of Ghana Medical School, P. O. Box 4236, Korle Bu, Accra; 6 Department of Surgery, University of Development Studies, School of Medicine and Health Sciences, Tamale-Techiman Road, Tamale

**Keywords:** trauma, injury, education, training, continuing professional development

## Abstract

Injury is a major cause of death and disability in Ghana. Strengthening care of the injured is essential to reduce this burden. Trauma continuing professional development (CPD) courses are an important component of strengthening trauma care. In many countries, including Ghana, their use needs to be more uniformly promoted. We propose lowcost strategies to increase the utilization of trauma CPD in Ghana, especially in district hospitals and higher need areas. These strategies include developing plans by regional health directorates and teaching hospitals for the regions for which they are responsible. Lists could be kept and monitored of which hospitals have doctors with which type of training. Those hospitals that need to have at least one doctor trained could be flagged for notice of upcoming courses in the area and especially encouraged to have the needed doctors attend. The targets should include at least one surgeon or one emergency physician at all regional or large district hospitals who have taken the Advanced Trauma Life Support (ATLS) (or locally-developed alternative) in the past 4 years, and each district hospital should have at least one doctor who has taken the Primary Trauma Care (PTC) or Trauma Evaluation and Management (TEAM) (or locally-developed alternatives) in the past 4 years. Parallel measures would increase enrollment in the courses during training, such as promoting TEAM for all medical students and ATLS for all surgery residents. It is important to develop and utilize more “home grown” alternatives to increase the long-term sustainability of these efforts,

**Funding:**

None

## Introduction

Injury is a major cause of death and disability. Globally it is estimated that injury results in 4,880,000 deaths per year, for a rate of 65 deaths / 100,000 / year. The rate is higher in low- and middle-income countries (69 deaths /100,000 / year) than in high-income countries (49 deaths / 100,000 / year). The rate is especially high in sub-Saharan Africa, at 85 deaths / 100,000 / year. In Ghana, injuries cause over 20,000 deaths annually, for a rate of 77 deaths / 100,000 / year).^1^ Strengthening care of the injured (trauma care) is essential to reduce this burden. Trauma care depends on having adequate human resources (skills, training, staffing), physical resources (equipment, supplies), and infrastructure.

In terms of training, the most important is the foundational training that doctors, nurses, and other health workers receive in medical school or residency / or fellowship training, nursing school, or other schools. However, briefer courses, such as one- to three-day continuing professional development (CPD) type courses can be important adjuncts.

These courses can impart a core body of knowledge and skills on the most critical parts of trauma care in a short period, thereby assuring that professionals who take these courses have a uniform, adequate, baseline level of skill for the care of the injured.

These courses also provide important updates on recent advances. In this article, the authors provide examples of the effectiveness and use of trauma CPD courses worldwide, review what is available for trauma CPD in Ghana, and present a proposal to increase the use and distribution of trauma CPD in Ghana.

### Effectiveness and use of trauma CPD courses

Many universities run occasional CPD courses on trauma care. Also, several professional societies have developed standardized courses, which are run in the same fashion in many countries. Some examples include (among others): Advanced Trauma Life Support (ATLS, run by the American College of Surgeons); National Trauma Management Course (NTMC, run by the International Association for Trauma Surgery and Intensive Care); Primary Trauma Care (PTC, run by the PTC Foundation); Trauma Evaluation and Management (TEAM, run by the American College of Surgeons); and Trauma Nursing Core Course (TNCC, run by the Emergency Nurses Association). NTMC is run primarily in India and Sri Lanka, but individual courses have been run in Kazakhstan and several African countries.^2^ ATLS is run in 80 countries, PTC in 70 countries, and TNCC in 14 countries.^3^–^5^

There is a solid evidence base showing the effectiveness of these CPD courses. One of the best studied of the courses is ATLS. A review in 2014 identified 23 articles that have been published on this topic in a wide range of countries, and for varied participant groups (including surgeons, emergency physicians, general doctors, and medical students). The studies consistently showed significant improvements in knowledge and skills for trauma care.^6^ Other studies have confirmed the benefits of such training in multiple countries.

For example, in Trinidad, the introduction of the ATLS course resulted in improvement in several processes of care measures (e.g. endotracheal intubation, IV resuscitation) and a decrease in mortality, among the most seriously injured group of patients, from 67% to 34%.^7^,^8^ In Spain, an increase in the percentage of doctors with ATLS training was associated with a decrease in the percentage of all trauma deaths that were preventable from 24% to 5% over 6 years. 9 In the Netherlands, the quality of multiple processes of care measures (e.g. airway maintenance) was found to be significantly improved after the introduction of the ATLS course in a study that utilized video recording of procedures.^10^

Given the documented effectiveness of trauma CPD courses, several countries have required their use in certain circumstances. For example, the American College of Surgeons has established criteria for human and physical resources that are required to be in place for hospitals of different levels that care for the injured in the USA and Canada.

One of these criteria concerns ATLS training. For example, the trauma medical director at a hospital is required to be currently credentialed in ATLS (have taken and passed it within the past four years). All surgeons taking trauma call must have taken ATLS at least once and must have 16 hours per year of any trauma CPD. Similar requirements pertain to other types of trauma care providers. These requirements are evaluated during the trauma centre verification visits carried out by the American College of Surgeons.^11^ Similar criteria exist in many other high-income countries.

Less stringent, but still significant requirements for trauma CPD exist in some low- and middle-income countries. For example, in South Africa, ATLS is required to write the specialty examination for surgery and emergency medicine.^12^,^13^ In Mexico, there are requirements on an institution-by-institution basis, especially for the larger trauma centres.^14^,^15^ In the absence of such efforts to require and promote trauma CPD, usage of such trauma CPD is less than optimal in many countries. Recruitment for enrolment for such courses is primarily done through informal word of mouth and occasional advertising. However, many hospitals have no doctor who has had this type of training.^15^

### What is available for trauma CPD in Ghana

There are currently several trauma CPD courses available in Ghana (Table 1). These include some of the courses noted above, such as ATLS, PTC, and TEAM. There are also occasional trauma-related courses organized by the universities and teaching hospitals. For example, the University of Cape Coast has run a 3-day course annually over the past 6 years. This has sought to improve outcomes in trauma care and has covered on-scene care and retrieval, in-patient management, mass casualty incidents, and lately, TEAM. An evaluation of these efforts showed the effectiveness of TEAM in improving participants' knowledge and, importantly, the retention of this knowledge several months after the course.^16^

**Table 1:** Trauma continuing professional development courses available in Ghana

Course	Usual participants	International organizer	Organizer in Ghana
**Advanced** **Trauma Life** **Support**	Doctors	American College of Surgeons	Ghana College of Physicians and Surgeons
**Orthopaedic** **courses**	Doctors	AO Foundation	Ghana College of Physicians and Surgeons
**Primary** **Trauma Care**	Anaesthetists, doctors, nurses, physician assistants, others	Primary Trauma Care Foundation	
**Trauma Evaluation** **and Management**	Doctors, medical students, nurses	American College of Surgeons	University of Cape Coast
**Individual local** **courses**	Variable		Universities and teaching hospitals

A survey of hospitals in Ghana showed that less than half of doctors who should have this type of training (i.e. doctors caring for the injured) had taken it 15. Likely, the levels of CPD training could be improved upon or at least use of the CPD courses could be more standardized. The ideal is that all doctors caring for the injured should have the skills taught in these courses. More consistent use of trauma CPD could also be an important adjunct to the efforts of the Ghana College of Physicians and Surgeons (GCPS) to increase the number of surgeons, emergency physicians, and other specialists who care for injured patients.^17^

### Proposal to increase coverage of trauma CPD courses

Currently, many hospitals, especially the smaller district hospitals, have no doctor who has had this type of training. The ideal is that all doctors who care for the injured should have had such a course, preferably at least once recently. However, much could be accomplished by having at least one doctor at each district hospital who has some form of the above training and by having several doctors with such training at each regional hospital and each large district hospital.

The above could be achieved by better planning. This could be done at both district and regional levels. Lists could be kept and monitored of which hospitals had doctors with which type of training. Those hospitals that need to have at least one doctor trained could be flagged for notice of upcoming courses in the area and especially encouraged to have the needed doctors attend. This could be done by the regional health directorates or by the teaching hospitals for the regions for which they are responsible.

Initially, a plan would be developed so that at least one of either a surgeon (member or fellow) or emergency physician (member or fellow) at all regional or large district hospitals would have taken ATLS in the past four years. Due to the high cost of ATLS, eventually lower cost, locally developed, but similar, alternatives could be substituted. An additional goal would be that each district hospital has at least one doctor who has taken PTC or TEAM in the past four years. ATLS would also count for this, as would other suitable locally developed courses.

Another goal would be that each district hospital has at least one senior nurse or emergency nurse who has taken PTC (or other suitable locally developed courses) in the past four years. Additional variations of the above plans could involve the AO orthopaedic care courses. Likewise, the needs of specific regions could be communicated to those organizing the ATLS, PTC, and TEAM courses, so that they could consider conducting courses in especially high-need areas.

The above plan would involve some effort on the part of a designated educational coordinator or other liaison affiliated to each of the regional health directorates or the teaching hospitals but would involve no additional funds. Doctors and nurses who take the courses would have the courses paid for as is currently done (a mix of courses sponsored by outside groups, some expenses paid for by participants' hospitals, and some expenses occasionally met by the participants).

If funds were available, the above plan could be facilitated by meeting part of the expenses, such as course fees. Such funds might especially be useful to assure sufficient training for more remote, lower-income areas. Hopefully, in the future, increased use of distance learning (e.g. online modules for pre-course materials) will reduce the time spent on the physically present components of the courses and thus lower their cost and make them more accessible.

Such efforts could also be aided by parallel measures to increase enrolment in the courses during training, as with the example from South Africa noted above. The GCPS is currently planning to promote TEAM widely for medical students and pre-residency doctors. Consideration is also being given to make ATLS compulsory for all surgery residents. In addition, senior residents in emergency medicine are currently being encouraged to become ATLS instructors.

## Conclusion

To assure long-term sustainability of efforts to increase use of trauma CPD, it is important to develop (and emphasize the use of) more “home grown” courses. For example, in the past, the Kwame Nkrumah University of Science and Technology (KNUST) developed such a home-grown trauma course, which ran annually in Kumasi. An evaluation of that course documented a high success rate in increasing knowledge of quality trauma care and a high rate of usage of the skills taught.^18^ Such locally developed courses are less expensive than the internationally run courses and would increase the availability of trauma CPD more widely in Ghana.

Efforts to develop and promote local courses would be aided by good evaluation, such as by following postcourse test results and by follow-up with doctors who have taken the courses to see how the courses have impacted their practice. We feel that the efforts recommended in this article would go a long way in strengthening the care of injured patients throughout Ghana.
